# Inactivation of Surface-Associated Viruses in Real Indoor Environments by a Humidification System Generating Vaporized Free Chlorine Components

**DOI:** 10.3390/microorganisms14040814

**Published:** 2026-04-02

**Authors:** Saki Kawahata, Mayumi Kondo, Atsushi Yamada, Naoya Shimazaki, Makoto Saito, Hiroyuki Tsukagoshi, Takayoshi Takano, Tetsuyoshi Yamada, Toshihiro Takei, Takashi Nakagawa, Miu Takada, Nobuhiro Saruki, Hirokazu Kimura

**Affiliations:** 1 Panasonic Ecology Systems Co., Ltd., Kasugai 486-8522, Japan; kawahata.saki@jp.panasonic.com (S.K.); takano.takayoshi@jp.panasonic.com (T.T.); yamada.tetsuyoshi@jp.panasonic.com (T.Y.); takei.toshihiro@jp.panasonic.com (T.T.); nakagawa.takashi003@jp.panasonic.com (T.N.); 2 Department of Clinical Engineering, Faculty of Medical Science and Technology, Gunma Paz University, Takasaki 370-0006, Japan; kondo@paz.ac.jp (M.K.); n-shimazaki@paz.ac.jp (N.S.); ma-saito@paz.ac.jp (M.S.); t.miumiu501@icloud.com (M.T.); 3 Gunma Prefectural Institute of Public Health and Environmental Sciences, Maebashi 371-0052, Japan; tsuka-hiro@pref.gunma.lg.jp (H.T.); saruki-n@pref.gunma.lg.jp (N.S.); 4 Department of Health Science, Graduate School of Health Science, Gunma Paz University, Takasaki 370-0006, Japan

**Keywords:** infection control, surface microorganisms, real indoor environment, vaporized free chlorine components, HOCl, hypochlorous acid, virus inactivation, protein structure analysis

## Abstract

Vaporized free chlorine, primarily present as hypochlorous acid (HOCl), is increasingly used for indoor microbial control; however, virus-dependent susceptibility and its molecular determinants remain unclear. We evaluated virucidal effects under controlled indoor conditions (0–9 ppb) against echovirus 30 (E30), influenza A/H1N1, and human adenovirus type 3 (HAdV3). Infectious titers were quantified by TCID_50_ assays. Computational fluid dynamics (CFD) simulations and gas-sensor measurements assessed spatial dispersion, and structural analyses examined oxidation-sensitive amino acid residues. Significant reductions in infectivity were observed for E30 (99.0%, *p* = 0.00727) and influenza A/H1N1 (99.9%, *p* = 0.000597), whereas no significant reduction was detected for HAdV3 (*p* = 0.142). Analyses including all data points without outlier exclusion confirmed the robustness of these findings. CFD indicated uniform dispersion, although spatial heterogeneity within the indoor environment cannot be excluded. These findings suggest that viral susceptibility to vaporized HOCl is associated with residue-level composition and structural context; however, this relationship should be interpreted as correlative rather than causal. Moreover, integration of molecular and structural analyses provides a plausible mechanistic framework, although direct biochemical validation remains necessary. Structural analyses showed lower proportions of oxidation-sensitive residues in adenoviral proteins compared with influenza A hemagglutinin (OR = 0.34–0.40, adjusted *p* < 0.001) and the E30 VP1 intermediate. Residues were clustered in surface-exposed functional domains in susceptible viruses.

## 1. Introduction

Microorganisms present in indoor environments constitute a persistent source of human exposure and play an important role in the transmission of infectious diseases [1]. In particular, frequently touched environmental surfaces can act as reservoirs for pathogenic microorganisms, facilitating indirect transmission through fomites and increasing the risk of contact-mediated infection [2]. This mode of transmission is relevant not only in domestic and communal living spaces but also in healthcare facilities, long-term care institutions, and educational settings, where environmental surface contamination has been implicated in outbreaks with substantial clinical and public health consequences [3].

Recent evaluations by international public health authorities have emphasized the importance of managing surface-associated microorganisms in indoor environments as a critical component of infection prevention and control strategies [4]. Accordingly, effective environmental interventions targeting contaminated surfaces are increasingly recognized as essential complements to established measures such as hand hygiene, personal protective equipment, and vaccination programs [5].

Environmental surface decontamination is commonly achieved through the application of liquid disinfectants. However, only a limited number of chemical agents exhibit broad-spectrum antimicrobial activity [6]. Moreover, widely used disinfectants, including sodium hypochlorite solutions and formaldehyde-based agents, are associated with notable drawbacks, such as mucosal irritation, corrosive effects on materials, and the potential release of harmful gases, which limit their suitability for continuous use in occupied indoor environments. In addition, surface disinfection relying on manual wiping procedures is inherently dependent on operator compliance and frequency of application, leading to variability in effectiveness [7]. These limitations highlight the need for non-contact, continuously operating environmental control strategies that can be safely applied in occupied indoor spaces.

In response to these limitations, low-concentration gaseous and/or vaporized disinfectants have emerged as a promising alternative for the non-contact and continuous control of surface-associated microorganisms [8]. Vaporized hypochlorous acid (HOCl) has been reported to inactivate a broad range of microorganisms, including bacteria and fungi, at concentrations considered safe for human exposure [9,10,11,12,13,14,15,16,17]. Similar antimicrobial effects at low concentrations have also been demonstrated for chlorine dioxide gas [18]. These findings suggest that oxidative gaseous agents may offer a practical approach to surface decontamination while avoiding many of the constraints associated with conventional liquid disinfectants. However, most previous studies have primarily focused on empirical antimicrobial efficacy, and virus-dependent differences in susceptibility under environmentally relevant indoor conditions remain insufficiently characterized.

At the molecular level, HOCl is a highly reactive electrophilic oxidant generated physiologically by the myeloperoxidase–hydrogen peroxide–chloride system during innate immune responses. Owing to its strong electrophilic characteristics, HOCl preferentially reacts with nucleophilic amino acid side chains, particularly sulfur-containing residues such as cysteine and methionine, as well as histidine, tyrosine, tryptophan, and lysine. These reactions result in oxidative modification, chlorination, and structural destabilization of proteins, potentially leading to loss of biological function. While these biochemical properties provide a plausible basis for viral inactivation, direct experimental evidence linking residue-level chemical reactivity to virus-specific susceptibility under low-concentration gaseous exposure remains limited. In addition, environmental parameters such as relative humidity and airflow dynamics are known to influence both viral persistence and the efficacy of gas-phase disinfectants [9,17], underscoring the importance of evaluating virucidal effects under realistic indoor environmental conditions rather than strictly controlled laboratory settings.

We previously developed a composite environmental control system combining a generator of vaporized free chlorine species with a fine-particle removal filter. Previous studies demonstrated that this system effectively reduced environmental bacterial contamination in real-world settings and inactivated bacteria inoculated onto plastic surfaces under laboratory conditions [19]. While these investigations established the antibacterial performance of the system, its virucidal efficacy—particularly against surface-associated viruses relevant to contact transmission—has not yet been comprehensively evaluated. Furthermore, the molecular determinants underlying virus-dependent susceptibility to low-level oxidative exposure remain insufficiently understood. Integrating experimental virology with computational and structural approaches provides a useful framework for addressing this gap. Quantitative infectivity assays enable direct evaluation of viral inactivation, while computational fluid dynamics (CFD) simulations can characterize the spatial distribution of disinfectant exposure. In parallel, comparative analysis of viral protein composition and three-dimensional structural mapping of chemically reactive residues can offer mechanistic insights. However, such analyses are inherently inferential and should be interpreted as providing a plausible mechanistic framework rather than definitive biochemical proof.

In the present study, we investigated the virucidal activity of vaporized free chlorine components (HOCl) using three clinically important viruses: human adenovirus type 3 (HAdV3), echovirus type 30 (E30), and influenza A virus (A/H1N1)pdm09 (AH1pdm09). These viruses were selected as representative non-enveloped and enveloped viruses associated with environmental persistence and indirect transmission. Notably, all viruses used in this study were clinical isolates rather than laboratory-adapted prototype strains, thereby enhancing the relevance of the findings to real-world indoor environments.

We hypothesized that virus-dependent susceptibility to vaporized HOCl is influenced by residue-level chemical composition and structural accessibility of viral surface proteins. Specifically, differences in the abundance and spatial distribution of HOCl-reactive amino acid residues may affect the likelihood that low-concentration oxidative exposure leads to functionally disruptive structural modification. Accordingly, this study aimed not only to evaluate the virucidal efficacy of vaporized hypochlorous acid under real indoor conditions but also to explore potential molecular determinants underlying differential virus inactivation, while providing a mechanistic interpretation that remains to be further validated by direct biochemical approaches.

## 2. Materials and Methods

### 2.1. Experimental Setup for Surface Viral Inactivation Under Vaporized Free Chlorine Exposure

Surface-associated viral inactivation experiments were carried out in a practical training laboratory at Gunma Paz University. The room volume was 260 m^3^ (10 m × 9.3 m × 2.8 m) and included two air-conditioning outlets. Although three ventilation openings were present, the ventilation system was intentionally disabled to prevent external air exchange. Ambient temperature and relative humidity were controlled using an air-conditioning unit and a steam humidifier (T-fal Groupe SEB Japan K.K., Tokyo, Japan), maintaining ranges of 18.6–23.3 °C and 45–76%, respectively. All procedures were performed under unoccupied conditions. For the control setup, samples were placed within a sealed 16 L plastic container (Iwasaki Industry Inc., Yamato-Koriyama, Japan), where temperature and humidity were maintained at 18.8–23.3 °C and 15–72%, respectively. Two experimental devices were installed on one side of the laboratory and operated continuously for 24 h. Vaporized free chlorine component levels were continuously measured both at the device outlet and at the sample location at 10 s intervals using a chlorine gas detector (New Cosmos Electric Co., Ltd., Osaka, Japan), with values converted into equivalent chlorine gas concentrations. Environmental temperature and humidity at the sample location were recorded every 60 s by a Thermo Recorder (Sato Shouji Inc., Kawasaki, Japan). Although the reported environmental ranges represent aggregated variability across experiments, intra-experimental fluctuations within each 24 h period remained limited and stable.

### 2.2. Generation of Vaporized Free Chlorine Components

The system used in this study produced humidified airflow by passing air through a rotating fibrous medium saturated with electrolyzed water. Electrolyzed water was generated by dissolving sodium chloride tablets in tap water, followed by in situ electrolysis within the device. Hypochlorous acid, the primary active species, volatilized concurrently with water vapor and was released into the surrounding air as vaporized free chlorine components. Electrolyzed water containing approximately 100 mg/L of available chlorine was supplied to the rotating filter, and airflow was maintained at 5.6 m^3^/min. This concentration (<100 mg/L) was selected based on prior optimization and occupational safety considerations to ensure adequate generation efficiency while maintaining safe exposure levels [20]. Available chlorine concentrations were quantified using a chlorine meter (Kasahara Rika Kogyo Co., Ltd., Kuki, Japan). Preliminary validation experiments were conducted to define appropriate operational concentrations of vaporized free chlorine components. While the occupational exposure threshold for chlorine gas is 500 ppb, no formal standard exists for vapor-phase free chlorine components. Given that medical air sterilization systems typically operate below 0.1 ppm (100 ppb), the present system was designed within this safety margin. Continuous monitoring confirmed that operation of two devices within the 260 m^3^ laboratory for 24 h maintained airborne concentrations at ≤9 ppb. These conditions were therefore adopted for subsequent viral inactivation experiments.

### 2.3. Viral Materials and Inactivation Assay

**Figure 1:**
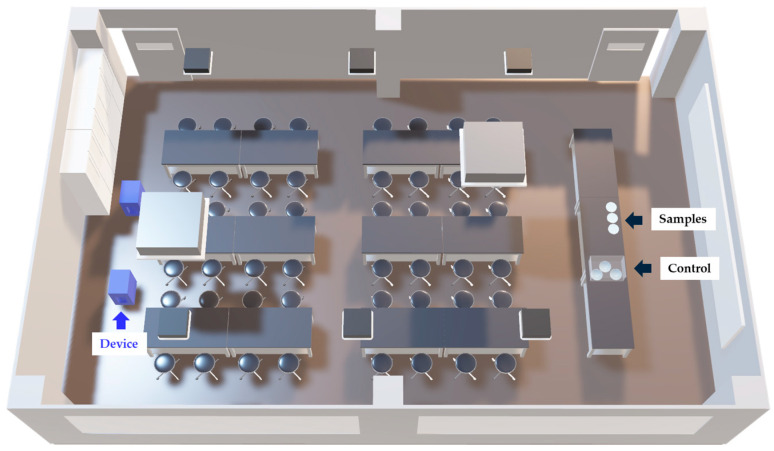
Schematic Overview of the Laboratory. The contact-based viral inactivation experiments were conducted in a university practical training laboratory. Desks and chairs were arranged in the central area of the room. Two experimental devices, indicated in blue in the figure, were installed side by side at the rear of the laboratory. Viral suspensions were applied onto culture dishes and used for the experiments. Dishes assigned to the Electrolyzed (+) condition were placed on an instructor’s desk located at the front of the room at a height of 0.8 m above the floor. For the control condition [Electrolyzed (−)], the dishes were placed inside a sealed plastic container and positioned on the same instructor’s desk. The distance between the placement site of the culture dishes and the experimental devices was approximately 8.0 m. This figure is adapted from reference [19], Kawahata et al., Microorganisms (2025), distributed under the terms of the Creative Commons Attribution (CC BY 4.0) license.

Three clinical viral isolates were used: human adenovirus type 3 (Ad3; Gunma/R6-108), echovirus 30 (E30; Gunma/H20-216), and influenza A virus (A/H1N1)pdm09 (A/Gunma/297/2024). Ad3 and E30 were propagated in HEp-2 and RD cells, respectively, whereas A/H1N1pdm09 was cultured in MDCK cells. Specifically, HEp-2 (ATCC CCL-23), RD (ATCC CCL-136), and MDCK (ATCC CCL-34) cell lines were used for virus propagation. All isolates were obtained from the Gunma Prefectural Institute of Public Health and stored at −80 °C until use. Immediately prior to experimentation, viral stocks were thawed, and 100 µL aliquots were applied onto culture dishes (AB2000, EIKEN CHEMICAL Co., Ltd., Chiyoda, Japan). An overview of the experimental setup is shown in Figure 1. Samples were positioned 0.8 m above the floor and 8.0 m from the operating devices (Electrolyzed (+) condition). For the control condition (Electrolyzed (−)), samples were placed inside a sealed 16 L plastic container, designated as Electrolyzed (−). To replicate drying conditions while excluding vaporized chlorine exposure, silica gel (1.0 g) and a low-voltage fan (Panasonic Corporation, Osaka, Japan) were placed inside the container. The container was tightly sealed to eliminate external airflow. Comparable drying times between Electrolyzed (+) and Electrolyzed (−) conditions were confirmed in advance. This sealed container system was designed to standardize drying conditions while preventing exposure to vaporized free chlorine components, although it does not fully replicate open-room airflow dynamics and gas exchange conditions. The viral suspensions were simultaneously allowed to air-dry under identical environmental conditions during exposure to ensure uniform surface-associated conditions. After 24 h of exposure, all samples were recovered. To maximize recovery efficiency, the surface was gently rinsed and resuspended using a dry transport system. Each viral sample was resuspended in 1 mL of recovery medium (DMEM supplemented with 2% fetal bovine serum). From this suspension, 100 µL was transferred onto a culture dish and re-suspended using a dry transport system (25-806 1PR BT, Sugiyama-Gen Co., Ltd., Tokyo, Japan), then returned to the recovery medium. Viral titration was performed in 96-well plates. Five wells (*n* = 5) were assigned to each sample and control. Serial dilutions were prepared, and host cells were inoculated accordingly. Ad3 and E30 were incubated at 37 °C with 5% CO_2_, whereas A/H1N1pdm09 was incubated at 34 °C with 5% CO_2_. The 50% tissue culture infectious dose (TCID_50_) values were calculated based on the presence or absence of cytopathic effects (CPEs) in each well using the Kärber method. CPEs were independently evaluated by two researchers using light microscopy, and wells were classified as either CPE-positive or CPE-negative based on morphological alterations consistent with virus-induced cytopathology. The assessments were not performed under blinded conditions.

### 2.4. Computational Simulation of Free Chlorine Dispersion

To characterize the transport and spatial distribution of vaporized free chlorine components, time-resolved simulations were conducted using proprietary computational fluid dynamics (CFD) technology. Simulations were performed with a three-dimensional structured grid model (STREAM V2025.1), incorporating airflow velocity, direction, and outlet concentration, as well as air-conditioning parameters. The diffusion coefficient was set to 1.21 × 10^−5^ m^2^/s. Processes contributing to concentration decay—including wall adsorption, ventilation removal, and chemical decomposition—were incorporated into the transient model. The governing equations included mass conservation and Navier–Stokes equations, with turbulence represented using the standard k–ε model. Mesh resolution was defined with a base size of 80 mm, with localized refinement in regions exhibiting steep gradients. Boundary conditions included inflow/outflow parameters consistent with experimental conditions and no-slip wall boundaries. Temporal concentration changes were visualized using contour mapping to illustrate spatial distribution dynamics. These loss processes were individually parameterized based on experimentally measured values [21].

### 2.5. Three-Dimensional Antigenic Structure and Mapping of Hypochlorous Acid-Reactive Amino Acid Residues

To elucidate the molecular basis of microbial inactivation by hypochlorous acid (HOCl), it is essential to characterize chemical modifications at the amino acid level [22]. HOCl preferentially reacts with susceptible side chains—particularly cysteine, methionine, histidine, tyrosine, tryptophan, and lysine—leading to oxidative modification, chlorination, and structural destabilization of antigenic proteins [23]. Mapping these reactive residues onto three-dimensional (3D) antigenic structures enables mechanistic interpretation of protein denaturation, epitope alteration, and potential loss of infectivity. Representative surface antigen proteins of the target microorganisms were selected based on their structural and immunological relevance. Based on the selected template sequences, three-dimensional structural models were constructed using Modeller version 10.4 [24]. The corresponding Protein Data Bank (PDB) identifiers and GenBank accession numbers of all template structures are provided in Supplementary Table S1 to ensure reproducibility. The generated models were subsequently inspected and evaluated using WinCoot (version 0.9.8.93) [25], and the model exhibiting the most favorable scoring metrics was selected as the optimal structure. Further structural refinement was performed through energy minimization using Swiss-PdbViewer (version 4.1.0) [26]. Structural quality was assessed prior to downstream analysis. HOCl-reactive amino acid residues were identified based on established chemical susceptibility profiles reported in the literature. Residue-specific reactivity was annotated according to known oxidation and chlorination pathways. The spatial distribution of these residues was analyzed to determine their localization within functional domains, receptor-binding regions, and antigenic epitopes. Three-dimensional visualization and residue mapping were performed using PyMOL (version 2.5.8) (Schrödinger, LLC, New York, NY, USA) [27]. Reactive amino acid residues were highlighted using color-coded surface and cartoon representations to distinguish highly susceptible residues from less reactive or structurally buried residues. Structural regions corresponding to antigenic epitopes were annotated where experimentally defined or inferred from prior reports. This integrative structural mapping approach enabled the correlation of HOCl-induced chemical reactivity at the amino acid level with conformational integrity of antigenic proteins, thereby providing mechanistic insight into microbial inactivation.

### 2.6. Statistical Analysis

Statistical analyses of viral infectivity were performed using EZR version 4.3.1 (Easy R; Jichi Medical University, Shimotsuke, Japan). Because viral titers typically follow a log-normal distribution, data were transformed to log_10_ values for descriptive analyses and outlier assessment. Outliers were identified using the interquartile range (IQR) method, in which values falling below Q1 − 1.5 × IQR or above Q3 + 1.5 × IQR on the log_10_ scale were considered extreme and excluded from subsequent analyses. However, given the relatively small sample sizes (*n* = 5–9), additional analyses including all data points without outlier exclusion were performed to assess robustness. Both analyses (with and without outlier exclusion) were compared, and consistent results were obtained. Group comparisons were conducted using the Mann–Whitney U test. The proportion of the target amino acid residues in each viral protein was calculated as the number of specific residues divided by the total number of amino acid residues in the corresponding protein. Differences in proportions among the four viral proteins were evaluated using a generalized linear model (GLM) with a binomial distribution and logit link function. An omnibus likelihood ratio test was first performed to assess whether the proportions differed among proteins. When a significant overall difference was detected, pairwise comparisons were conducted using the GLM framework, and odds ratios (ORs) with 95% confidence intervals (CIs) were calculated. Multiple comparisons were adjusted using the Holm method. Confidence intervals were calculated using the Wilson score method. Statistical significance was defined as *p* < 0.05.

### 2.7. Ethics Status

The viral strains used in this study were clinical isolates obtained through the National Epidemiological Surveillance of Infectious Diseases (NESID) program in Japan, conducted under the Act on the Prevention of Infectious Diseases and Medical Care for Patients with Infectious Diseases (Infectious Diseases Control Law). These specimens were collected as part of routine public health surveillance and were not newly collected for research purposes. All samples were fully anonymized prior to their use, and no personally identifiable information was included. Accordingly, this study represents the secondary use of surveillance-derived specimens. The use of such specimens for research purposes is conducted in accordance with the Infectious Diseases Control Law and the Ethical Guidelines for Medical and Health Research Involving Human Subjects in Japan. Within this framework, the requirement for written informed consent was waived by the Medical Research Ethics Committee of Gunma Paz University, and verbal informed consent was obtained as appropriate within routine public health surveillance practices. This study was reviewed and approved by the Medical Research Ethics Committee of Gunma Paz University (Approval No. PAZ25-50, approved on 25 February 2026).

## 3. Results

### 3.1. Surface Virucidal Activity of Vaporized Free Chlorine Components

The infectious titers (TCID_50_/mL) obtained for each virus under Electrolyzed (−) and Electrolyzed (+) conditions are summarized in Table 1, together with percentage reductions and statistical comparisons. For echovirus 30 (E30), the mean infectious titer decreased from 8.3 × 10^4^ TCID_50_/mL (*n* = 5) under the Electrolyzed (−) condition to 8.0 × 10^2^ TCID_50_/mL (*n* = 6) under the Electrolyzed (+) condition, corresponding to a significant 99.0% reduction (*p* = 0.00727). One value in the Electrolyzed (−) group exceeded the interquartile range (IQR) threshold on the log_10_ scale and was excluded. Inclusion of all values yielded consistent statistical outcomes (*p* = 0.00462), as detailed in Supplementary Table S2. During this experiment, vaporized free chlorine concentrations averaged 19.6 ppb (range: 12–29 ppb) at the device outlet and 3.3 ppb (range: 0–7 ppb) at the sample location. For influenza A (A/H1N1)pdm09, the infectious titer declined from 3.0 × 10^5^ TCID_50_/mL (*n* = 9) in the Electrolyzed (−) condition to 4.1 × 10^2^ TCID_50_/mL (*n* = 8) in the Electrolyzed (+) condition, representing a 99.9% reduction (*p* = 0.000597). One outlier was identified and excluded in the Electrolyzed (+) group based on the IQR criterion. Reanalysis, including all data points, did not alter statistical significance (*p* = 0.00039). The mean vaporized free chlorine concentration was 14.0 ppb (range: 7–27 ppb) at the outlet and 2.0 ppb (range: 0–4 ppb) at the sample site.

In contrast, for human adenovirus type 3 (Ad3), infectious titers were 6.9 × 10^4^ TCID_50_/mL (*n* = 9) under the Electrolyzed (−) condition and 4.1 × 10^4^ TCID_50_/mL (*n* = 8) under the Electrolyzed (+) condition, showing no statistically significant difference (*p* = 0.142). One outlier was removed from the Electrolyzed (+) dataset; however, inclusion of all observations did not change the outcome (*p* = 0.287). Vaporized free chlorine concentrations averaged 15.2 ppb (range: 9–24 ppb) at the outlet and 3.5 ppb (range: 0–9 ppb) at the sample site. Overall, these findings indicate that vaporized free chlorine—predominantly consisting of hypochlorous acid—markedly reduces surface-associated E30 and influenza A virus under realistic indoor conditions, even at distances of approximately 8.0 m from the emission source. By contrast, the antiviral effect against Ad3 was limited under identical conditions. These observations should be interpreted within the constraints of the experimental setup and may not fully generalize to all indoor environments.

### 3.2. Simulation Analysis of Vaporized Free Chlorine Components

**Figure 2:**
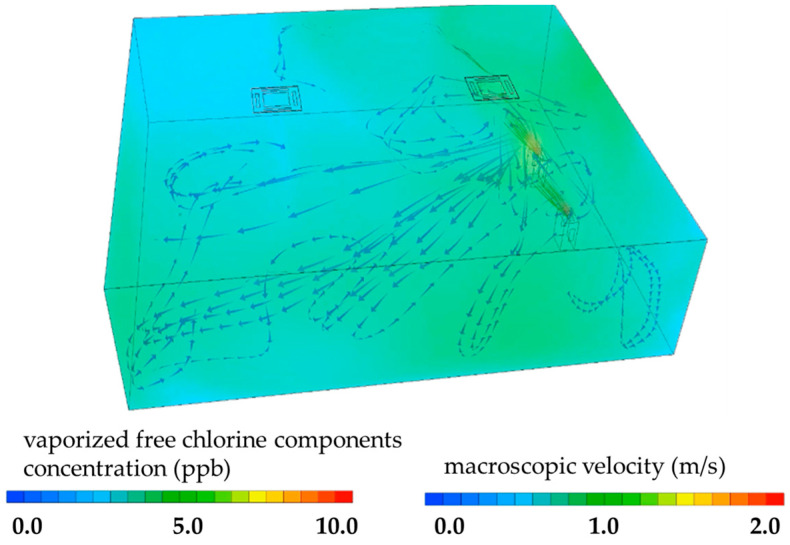
Simulation Analysis of Vaporized Free Chlorine Components. The figure presents the simulated concentration distribution of vaporized free chlorine and airflow dynamics 1200 s after activation of the device. The concentration of vaporized free chlorine is represented using color mapping. The direction of the humidified airflow emitted from the device is indicated by arrows, and airflow velocity is visualized by the color gradient of the arrows.

The spatial distribution and transport behavior of vaporized free chlorine within the experimental space were assessed using computational fluid dynamics (CFD), as illustrated in Figure 2. Simulation results indicated that approximately 1200 s after device activation, the vaporized free chlorine had dispersed throughout the room and approached a steady-state concentration profile. The concentration contours demonstrated that the gaseous components reached locations up to ~8.0 m from the device. These simulated distributions were consistent with empirical measurements obtained from gas sensors at the sample sites (0–9 ppb), supporting the validity of the CFD-based diffusion model under the tested indoor conditions. Although both the simulation and measurement indicate broad spatial coverage, the presence of localized variability in concentration cannot be completely excluded.

### 3.3. Structural Basis for Differential Viral Susceptibility to HOCl

**Figure 3:**
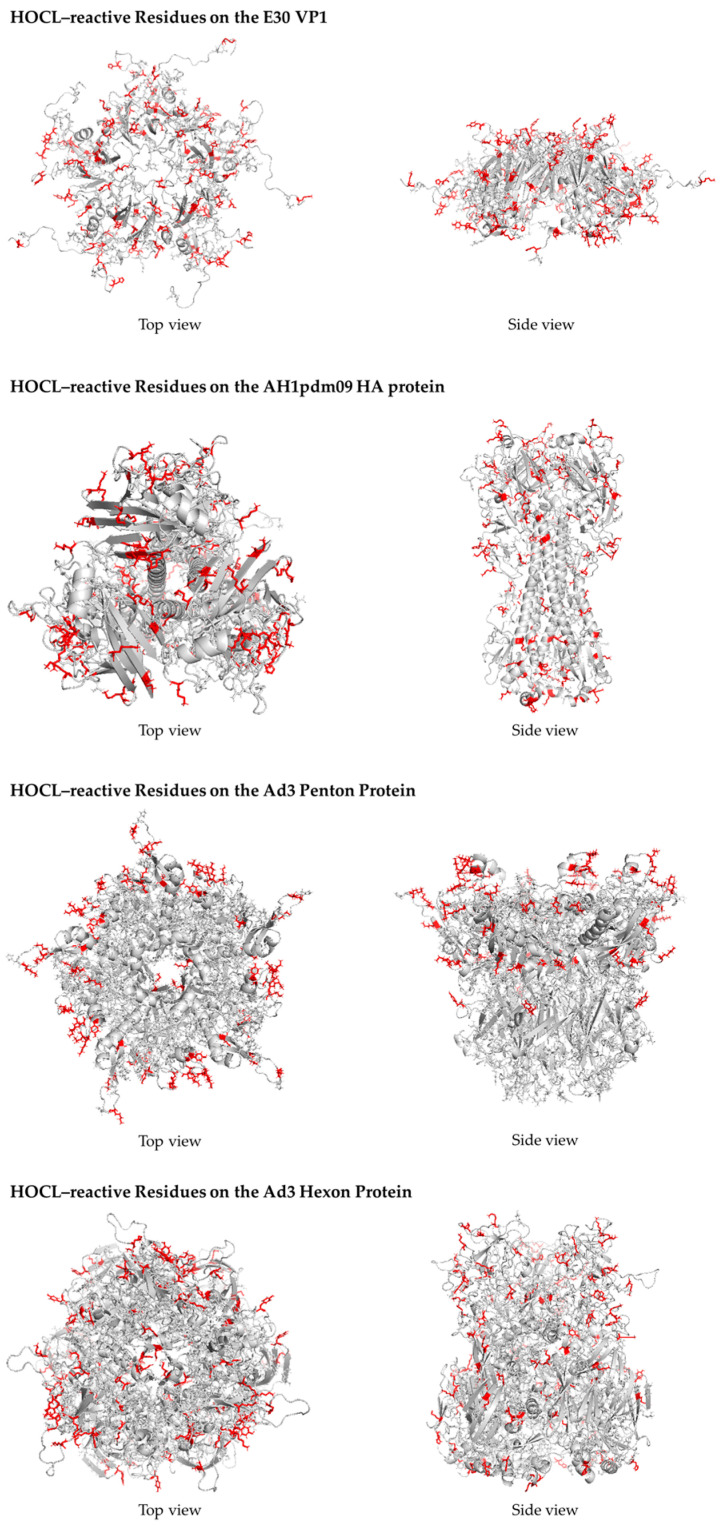
Three-Dimensional Antigenic Structure and Mapping of Representative Hypochlorous Acid-Reactive Amino Acid Residues. Three-dimensional structural representations of viral antigenic proteins are shown for echovirus 30 (E30) VP1, influenza A(H1N1)pdm09 hemagglutinin (HA), and human adenovirus type 3 (Ad3) penton and hexon proteins. Amino acid residues predicted to be chemically reactive with hypochlorous acid (HOCl)—including cysteine (Cys), methionine (Met), histidine (His), tyrosine (Tyr), tryptophan (Trp), and lysine (Lys)—are highlighted in red. These residues represent oxidation-susceptible side chains that are preferential targets of HOCl-mediated modification. Comparative visualization of their spatial distribution reveals protein-specific differences in surface exposure and localization within antigenically or functionally important domains. Structural mapping was performed to explore potential molecular determinants underlying virus-dependent susceptibility to HOCl-mediated inactivation.

To investigate whether differences in protein composition contribute to variability in virucidal susceptibility, the relative abundance of hypochlorous acid (HOCl)-reactive amino acid residues was analyzed in representative viral antigenic proteins (Figure 3). To further elucidate the structural and positional determinants of hypochlorous acid (HOCl) susceptibility at the amino acid side-chain level, monomeric structures of each antigenic protein were analyzed (Supplementary Figure S1). The proportions of reactive residues were calculated as follows: 2.52% (56/2220) in the HAdV3 penton protein, 2.95% (83/2812) in the HAdV3 hexon protein, 5.81% (66/1136) in the E30 VP1 protein, and 7.03% (105/1494) in the influenza A hemagglutinin (HA) protein. These quantitative data are summarized in Table 2. A generalized linear model (GLM) revealed a significant overall difference among the four proteins (χ^2^(3) = 49.7, *p* < 0.0001). Using influenza A HA as the reference, the odds of containing HOCl-reactive residues were significantly lower in HAdV3 penton (OR = 0.34, 95% CI: 0.25–0.46, adjusted *p* < 0.001) and hexon proteins (OR = 0.40, 95% CI: 0.31–0.53, adjusted *p* < 0.001). In contrast, E30 VP1 showed a non-significant reduction (OR = 0.80, 95% CI: 0.62–1.04, adjusted *p* = 0.12). Pairwise comparisons indicated no statistically significant difference between penton and hexon (OR = 0.85, adjusted *p* = 0.36), nor between E30 VP1 and influenza A HA. From a structural perspective, adenoviral penton and hexon proteins form a rigid icosahedral capsid framework, whereas E30 VP1 and influenza A HA represent surface-exposed antigenic proteins directly interacting with the extracellular environment. The lower proportion of reactive residues in adenoviral proteins suggests a reduced density of chemically susceptible sites within the capsid architecture. Conversely, the higher proportions observed in E30 and influenza A proteins indicate increased availability of oxidation-prone residues within exposed domains. These compositional differences may contribute to differential susceptibility to HOCl-mediated oxidative modification. However, this relationship remains correlative and does not establish a direct mechanistic pathway.

**Table 2:** Comparative Proportion of HOCl-Reactive Amino Acid Residues Among Three Viruses.

Virus	Antigens	Reactive Residues (*n*/*N*)	%	95% CI (Wilson)	OR vs. HA	95% CI	*p* Value	OR vs. VP1	95% CI	*p* Value
E30	VP1	66/1136	5.81	4.59–7.32	0.80	0.62–1.04	0.12	Reference	—	—
A/H1N1	HA	105/1494	7.03	5.84–8.44	Reference	—	—	1.25	0.96–1.61	0.10
Ad3	Penton	56/2220	2.52	1.95–3.26	0.34	0.25–0.46	<0.001	0.42	0.29–0.60	<0.001
	Hexon	83/2812	2.95	2.39–3.64	0.40	0.31–0.53	<0.001	0.49	0.35–0.68	<0.001

Proportions represent the number of representative HOCl-reactive amino acid residues (Cys, Met, His, Tyr, Trp, and Lys) divided by the total number of amino acid residues in each respective protein. Confidence intervals for the proportions were calculated using the Wilson score method. Influenza A hemagglutinin (HA) and E30 VP1 were used as reference categories for the respective odds ratio comparisons. *p* values for comparisons versus influenza A HA were adjusted for multiple testing using the Holm method. Ad3 denotes human adenovirus type 3; E30 denotes echovirus type 30; AH1 denotes influenza A (A/H1N1)pdm09. Percent values (%) indicate the proportion of HOCl-reactive amino acid residues.

## 4. Discussion

The present study demonstrates that vaporized free chlorine, primarily in the form of hypochlorous acid (HOCl), exerts virus-dependent inactivation effects under environmentally relevant indoor conditions. Significant reductions in infectious titers were observed for echovirus 30 (E30) and influenza A/H1N1, whereas no statistically significant reduction was detected for human adenovirus type 3 (HAdV3). Computational fluid dynamics (CFD) simulations and gas-sensor measurements indicated that vaporized free chlorine was broadly distributed throughout the experimental room, reaching locations approximately 8.0 m from the device. These findings suggest that the observed differences in virucidal efficacy are unlikely to be solely attributable to insufficient exposure at the sampling location; however, spatial heterogeneity within the indoor environment, including potential dead zones and localized airflow variations, cannot be completely excluded.

A key finding of this study is that viral susceptibility to vaporized HOCl cannot be explained solely by the presence or absence of a lipid envelope. While influenza A virus is enveloped and may be susceptible to oxidative disruption of membrane lipids, echovirus 30—an unenveloped virus—also exhibited substantial inactivation. In contrast, human adenovirus type 3, which is likewise non-enveloped, demonstrated relative resistance. These observations indicate that envelope status alone is insufficient to explain the differential susceptibility and suggest that intrinsic structural and molecular features of viral particles play a critical role.

One plausible explanation for these differences lies in the chemical reactivity of viral proteins. HOCl is a highly reactive electrophilic oxidant that preferentially modifies nucleophilic amino acid side chains, including cysteine, methionine, histidine, tyrosine, tryptophan, and lysine [22,23]. Oxidative modification of these residues can lead to alterations in protein conformation, disruption of intermolecular interactions, and loss of functional activity, including receptor binding and membrane fusion. In the present study, adenoviral penton and hexon proteins exhibited significantly lower proportions of HOCl-reactive residues compared with influenza A hemagglutinin, whereas E30 VP1 showed intermediate values. These compositional differences are consistent with the observed pattern of viral susceptibility. However, it is important to emphasize that this relationship should be interpreted as correlative rather than demonstrating a direct causal mechanism linking residue composition to viral inactivation.

Structural considerations further support this interpretation. Adenoviruses possess a highly stable icosahedral capsid composed of hexon and penton proteins arranged in a rigid lattice structure, which may confer resistance to chemical perturbation. In contrast, influenza A hemagglutinin and enteroviral VP1 are surface-exposed proteins that undergo dynamic conformational rearrangements during viral entry. Three-dimensional structural mapping in this study demonstrated that HOCl-reactive residues in HA and VP1 tend to cluster in surface-accessible and functionally relevant regions, whereas such residues are less abundant and more structurally constrained in adenoviral capsid proteins. These structural observations provide a plausible mechanistic framework for differential susceptibility; however, they do not constitute direct experimental proof of the inactivation mechanism.

An important practical implication of this study is that substantial viral inactivation was achieved at airborne concentrations in the low parts-per-billion range, which is well below established occupational exposure limits for chlorine gas [20]. This finding suggests that vaporized HOCl may represent a feasible approach for continuous environmental control of pathogens in occupied indoor environments, balancing antimicrobial efficacy with safety considerations.

The present study was intentionally designed to evaluate virucidal effects under realistic indoor environmental conditions rather than strictly controlled laboratory settings. Accordingly, environmental parameters such as relative humidity (RH) were allowed to vary within practical ranges. RH is known to influence both viral persistence on surfaces and the efficacy of gas-phase disinfectants [9,17], and therefore represents a potential confounding factor. In this study, RH was continuously monitored and remained relatively stable within individual experimental runs, although variation was observed across experiments. Because both Electrolyzed (+) and Electrolyzed (−) conditions were conducted under comparable ambient environmental conditions except for HOCl exposure, the relative differences in viral inactivation are unlikely to be explained solely by RH variation. Nevertheless, interactions between RH and HOCl-mediated virucidal activity cannot be fully excluded and warrant further investigation.

The experimental control design represents an additional consideration. To standardize drying conditions while preventing exposure to vaporized free chlorine components, samples in the control group were placed within a sealed container containing silica gel and a fan. This approach was adopted to minimize variability in moisture-dependent viral decay and to isolate the effect of HOCl exposure. However, the sealed micro-environment differs from an open indoor space in terms of airflow dynamics, gas exchange, and potential accumulation of volatile compounds. Therefore, this control condition may not fully replicate natural baseline conditions in an open room. A control experiment performed in the same room with the device turned off would represent an ideal comparator and should be considered in future studies.

Another limitation relates to spatial representation. Viral samples were evaluated at a single location within the room (8.0 m from the device at a height of 0.8 m). While CFD simulations and gas-sensor measurements indicated broad dispersion of vaporized free chlorine, the absence of multi-point validation limits the ability to generalize these findings to the entire indoor environment. Real indoor spaces are characterized by complex airflow patterns, including recirculation zones and areas of reduced mixing, which were not fully captured in the present design. Therefore, the current results should be interpreted as a proof-of-concept demonstrating effective viral inactivation at a representative distant point rather than definitive evidence of uniform whole-room efficacy.

Statistical considerations also merit attention. Although outliers were identified using the IQR method, additional analyses, including all data points without outlier exclusion, yielded consistent results, with statistically significant differences for E30 and A/H1N1 preserved. These findings support the robustness of the conclusions; however, the relatively small sample sizes (*n* = 5–9) remain a limitation and necessitate cautious interpretation.

Moreover, while the integration of virological assays, structural modeling, and statistical analysis provides a comprehensive framework for interpreting virus-dependent susceptibility, the proposed mechanism remains hypothetical and has not been directly validated at the biochemical level. Future studies employing techniques such as mass spectrometric identification of oxidized or chlorinated amino acid residues will be essential to confirm the molecular basis of HOCl-mediated viral inactivation and to establish causality [22,23].

In conclusion, this study demonstrates that vaporized HOCl exhibits virus-dependent inactivation under real indoor conditions and provides a structurally informed and chemically plausible explanation for differential susceptibility among viruses. However, the findings should be interpreted as supporting an inferential mechanistic model rather than definitive proof, and further experimental validation and environmental generalization are required.

## 5. Conclusions

This study demonstrates that vaporized free chlorine, primarily in the form of hypochlorous acid (HOCl), is associated with virus-dependent inactivation under environmentally relevant indoor conditions. Despite uniform dispersion at low parts-per-billion concentrations, significant reductions in infectivity were observed for echovirus 30 and influenza A/H1N1, whereas human adenovirus type 3 showed relative resistance. These differences cannot be explained solely by exposure conditions or the presence or absence of a viral envelope but instead appear to be influenced by intrinsic molecular and structural characteristics of viral proteins. Comparative analyses revealed that influenza A hemagglutinin and E30 VP1 contain higher proportions of oxidation-sensitive amino acid residues than adenoviral penton and hexon proteins. These findings suggest that residue-level chemical composition and structural context may contribute to differential susceptibilities to vaporized HOCl; however, this relationship should be interpreted as associative rather than demonstrating a definitive causal mechanism. Three-dimensional structural mapping further indicated that oxidation-prone residues in HA and VP1 are preferentially located within surface-exposed and functionally relevant domains, whereas adenoviral capsid proteins contain fewer such residues within rigid structural frameworks. These structural observations provide supportive evidence for a plausible mechanistic framework but do not constitute direct experimental proof of the inactivation mechanism. Taken together, the present results support a structure–chemistry–function paradigm in which viral sensitivity to vaporized HOCl is influenced not only by environmental exposure parameters but also by the abundance, accessibility, and spatial distribution of oxidation-sensitive residues within viral surface proteins. However, the proposed mechanism remains hypothetical and requires further validation through direct biochemical analyses. From a practical perspective, the observed virucidal effects at low ppb concentrations suggest that vaporized HOCl may represent a potentially useful approach for continuous environmental control in indoor settings while maintaining safety considerations. Nevertheless, extrapolation of these findings to diverse real-world environments should be undertaken with caution due to potential variability in environmental conditions and spatial heterogeneity. Future studies integrating biochemical identification of oxidative modifications, expanded viral panels, and multi-point environmental validation will be necessary to further elucidate the mechanisms and generalizability of HOCl-mediated viral inactivation.

## Figures and Tables

**Table 1:** Virucidal Effects of Vaporized Free Chlorine Components Upon Contact Exposure.

Virus	Electrolyzed (−) (TCID_50_/mL)	Electrolyzed (+) (TCID_50_/mL)	Reduction Rate (%)	*p* Value
E30	(8.3 ± 2.4) × 10^4^	(8.0 ± 3.4) × 10^2^	99.0 ± 0.4	0.00727
A/H1N1	(3.0 ± 3.7) × 10^5^	(4.1 ± 1.7) × 10^2^	99.9 ± 0.1	0.000597
Ad3	(6.9 ± 4.1) × 10^4^	(4.1± 1.7) × 10^4^	No significant	0.142

Data are expressed as mean ± standard deviation (SD). Detailed procedures are described in the text.

## Data Availability

The original contributions presented in this study are included in the article/Supplementary Material. Further inquiries can be directed to the corresponding author.

## Supplementary Materials

The following supporting information can be downloaded at: https://www.mdpi.com/article/10.3390/microorganisms14040814/s1, Figure S1: Enlarged structural visualization of HOCl-reactive amino acid residues in viral antigens. Monomeric structures of representative viral surface proteins from A/H1N1, Echovirus 30 (E30), and Human Adenovirus type 3 (HAdV3) are shown. Each structure is presented in an enlarged format to improve visualization of individual amino acid residues. All HOCl-reactive residues (e.g., Cys, Met, His, Tyr, Trp, and Lys) are highlighted in red and explicitly labeled within the structures. Enlarged views focus on surface-exposed regions, allowing clear identification of residue localization. These representations complement Figure 3 by providing higher-resolution structural detail and facilitate interpretation of the spatial distribution and clustering of oxidizable residues within viral antigens; Table S1: Template PDB identifiers used for structural modeling; Table S2: Statistical analysis including all data points for the virucidal effects of vaporized free chlorine components under contact exposure.
