# Supplementary material for: Inactivation of Surface-Associated Viruses in Real Indoor Environments by a Humidification System Generating Vaporized Free Chlorine Components
Source: Microorganisms. 2026 Apr 2;14(4):814. doi: 10.3390/microorganisms14040814 (PMC13118295; doi:10.3390/microorganisms14040814)
Supplement: Supplementary file 1 [file microorganisms-14-00814-s001.zip › microorganisms-4214659-supplementary.pdf]

Supplementary Materials

Supplementary Table S1. Template PDB identifiers used for structural modeling

Virus	Antigens	PDB ID	GenBank accession number
E30	VP1	6LHT	WHA31307.1
A/H1N1	HA	9GSP	PX663777.1
Ad3	Penton	2C6S	PV550961.1
	Hexon	7TAU	PV550961.1

Supplementary Table S2. Statistical analysis including all data points for the virucidal effects of vaporized free chlorine components under contact exposure.

Virus	Electrolyzed (–) ($TCID_{50}$/mL)	Electrolyzed (+) ($TCID_{50}$/mL)	Reduction Rate (%)	*p* Value
E30	$(7.1 \pm 3.4) \times 10^4$	$(8.0 \pm 3.4) \times 10^2$	98.9 ± 0.5	0.00462
A/H1N1	$(3.0 \pm 3.7) \times 10^5$	$(7.2 \pm 8.8) \times 10^2$	99.8 ± 0.3	0.00039
Ad3	$(6.9 \pm 4.1) \times 10^4$	$(5.0 \pm 3.1) \times 10^4$	No significant	0.287

Data are expressed as mean ± standard deviation (SD). Detailed procedures are described in the text.

Enlarged Structural Views of HOCl-reactive Residues on the E30 VP1

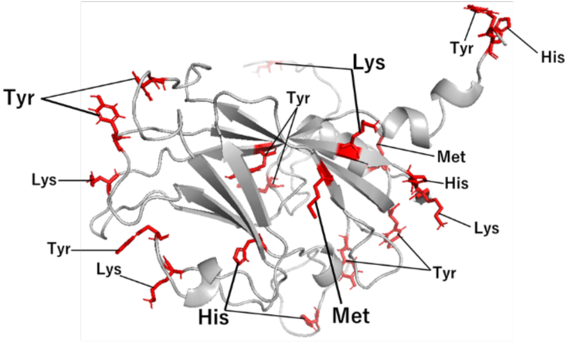

Enlarged Structural Views of HOCl-reactive Residues on the A/H1N1pdm09 HA Protein

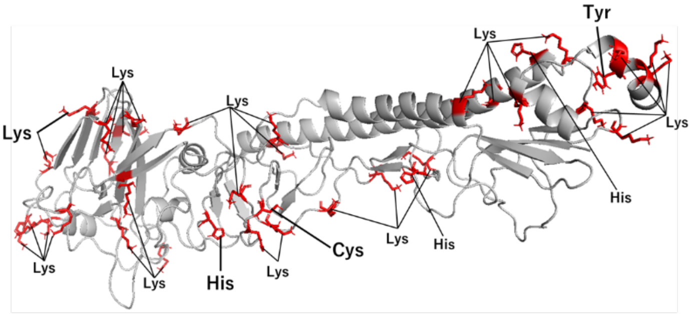

Enlarged Structural Views of HOCl-reactive Residues on the Ad3 Penton Protein

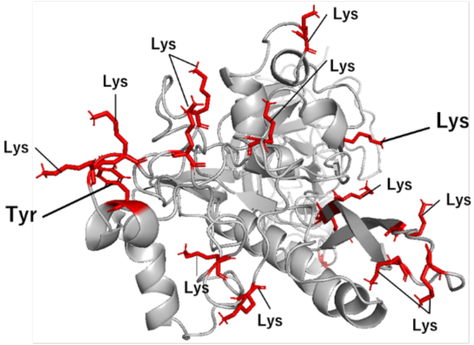

Enlarged Structural Views of HOCl-reactive Residues on the Ad3 Hexon Protein

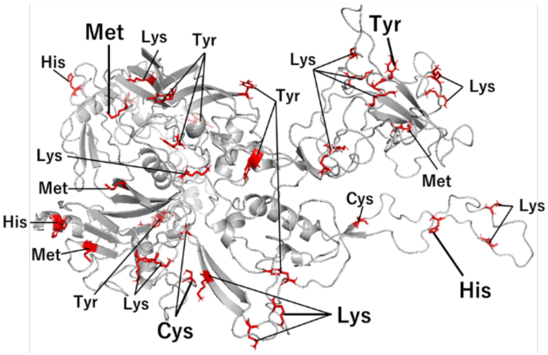

Supplementary Figure S1. Enlarged structural visualization of HOCl-reactive amino acid

residues in viral antigens. Monomeric structures of representative viral surface proteins from A/H1N1, Echovirus 30 (E30), and Human Adenovirus type 3 (HAdV3) are shown. Each structure is presented in an enlarged format to improve visualization of individual amino acid residues. All HOCl-reactive residues (e.g., Cys, Met, His, Tyr, Trp, and Lys) are highlighted in red and explicitly labeled within the structures. Enlarged views focus on surface-exposed regions, allowing clear identification of residue localization. These representations complement Figure 3 by providing higher-resolution structural detail and facilitate interpretation of the spatial distribution and clustering of oxidizable residues within viral antigens.
